# Fear of Killer Whales Drives Extreme Synchrony in Deep Diving Beaked Whales

**DOI:** 10.1038/s41598-019-55911-3

**Published:** 2020-02-06

**Authors:** Natacha Aguilar de Soto, Fleur Visser, Peter L. Tyack, Jesús Alcazar, Graeme Ruxton, Patricia Arranz, Peter T. Madsen, Mark Johnson

**Affiliations:** 1 BIOECOMAC. Dept. Animal Biology, Edaphology and Geology. University of La Laguna, Tenerife, Canary Islands Spain; 2 Department of Freshwater and Marine Ecology, IBED, University of Amsterdam/Department of Coastal Systems, NIOZ and Utrecht University/Kelp Marine Research, Hoorn, The Netherlands; 3 Sea Mammal Research Unit, University of St Andrews, St Andrews, Scotland UK; 4 School of Biology, University of St Andrews, St Andrews, Scotland UK; 5 Zoophysiology, Dept. of Bioscience, Aarhus University, Aarhus, Denmark

**Keywords:** Animal behaviour, Behavioural ecology

## Abstract

Fear of predation can induce profound changes in the behaviour and physiology of prey species even if predator encounters are infrequent. For echolocating toothed whales, the use of sound to forage exposes them to detection by eavesdropping predators, but while some species exploit social defences or produce cryptic acoustic signals, deep-diving beaked whales, well known for mass-strandings induced by navy sonar, seem enigmatically defenceless against their main predator, killer whales. Here we test the hypothesis that the stereotyped group diving and vocal behaviour of beaked whales has benefits for abatement of predation risk and thus could have been driven by fear of predation over evolutionary time. Biologging data from 14 Blainville’s and 12 Cuvier’s beaked whales show that group members have an extreme synchronicity, overlapping vocal foraging time by 98% despite hunting individually, thereby reducing group temporal availability for acoustic detection by killer whales to <25%. Groups also perform a coordinated silent ascent in an unpredictable direction, covering a mean of 1 km horizontal distance from their last vocal position. This tactic sacrifices 35% of foraging time but reduces by an order of magnitude the risk of interception by killer whales. These predator abatement behaviours have likely served beaked whales over millions of years, but may become maladaptive by playing a role in mass strandings induced by man-made predator-like sonar sounds.

## Introduction

Deep-diving marine mammals are expected to maximise time spent foraging in deep prey layers to offset the energetic and physiological costs of diving^1^. But Cuvier’s and Blainville’s beaked whales (*Ziphius cavirostris* and *Mesoplodon densirostris*, respectively) employ a diving behaviour unlike that of other deep-diving toothed whales: they restrict echolocation to the deepest part of long and deep foraging dives that are typically followed by extended series of shallower and silent non-foraging dives that result in less than 20% of time devoted to biosonar-mediated foraging^2–5^. Further, these species ascend slowly and silently from deep dives at a low pitch angle^2^. This unusual and costly diving style has been interpreted as serving to mitigate decompression sickness or to accommodate lactate build up from foraging dives that may exceed the aerobic dive limit^6^, but, see^7^. However, satisfactory physiological mechanisms to support these interpretations have yet to be found. When other toothed whales dive to similar depths, they do not display such a diving behaviour: both pilot whales that are similar in size to these beaked whales and the larger sperm whales ascend nearly vertically from their deep foraging dives^8,9^ and often emit calls during the ascent to mediate reunion with non-diving group members^10–13^. Because the highly stereotyped group diving and vocal behaviour of beaked whales is difficult to explain by foraging niche or physiology^2^ an alternate proposition is that it serves to abate predation risk^2,4,14^. Fear of predation can induce profound changes in the behaviour and physiology of prey species even if predator encounters are infrequent^15,16^. This is especially so for long-lived, slow-reproducing species such as whales for which even costly behaviours to abate predators have net fitness benefits^17^.

While beaked whales can be attacked by sharks^18^ and can be disturbed by delphinids^19^, their pre-eminent predators are killer whales^20–22^. The problem of avoiding predators with acute hearing such as killer whales is compounded for echolocating toothed whales that must make sound to find food^23^, making them detectable at long ranges by listening predators. In contrast, killer whales are often silent when hunting mammals, giving little advance notice of their presence^24^, and their power and speed^25^ limit the opportunities for last-minute escapes. Predation pressure from killer whales is thought to have driven some toothed whale species to vocalize beyond the hearing range of killer whales^26,27^, while large aggregations of other species, from dolphins to cohesive groups of sperm and pilot whales, provide social defence^28,29^. However, smaller species of beaked whales have adopted neither strategy: they produce medium frequency clicks^23^ that are detectable at considerable ranges^30^ and live in small groups that offer scant social defence^31^. This apparent lack of a predator abatement strategy is at odds with their intense reactions to playbacks of killer whale and mid-frequency sonar sounds: even sound exposure levels close to the ambient noise can cause intense behavioural responses in beaked whales^32–35^ suggesting that sonar-related mortalities^36,37^ are rooted in an extreme anti-predator response^38^. This leads us to posit that fear of predation is a major driver of the overall social and movement behaviour of beaked whales.

Here we test whether the distinctive features of beaked whale diving behaviour and group cohesiveness have quantitative benefits to reduce risk of predator encounters. We do so by analysing biologging data from Blainville’s and Cuvier’s beaked whales, that are among the best-known beaked whale species and also those most commonly found in mass strandings related to naval sonar^39^. We propose that fear of predation shapes the minute-by-minute behaviour of these long lived, elephant-sized marine mammals which pay this heavy cost to access a privileged foraging niche and mitigate interception by a stealthy large-brained cosmopolitan predator.

## Results and Discussion

We used biologging data from sound and movement recording tags^40^ on beaked whales, together with data reported in the literature, to quantify the predator abatement benefit of two aspects of their behaviour: (i) diving and vocal coordination, and (ii) ascent swimming.

### Coordination

Killer whales are large brained and muscular predators with a limited diving capacity^25,41^. Although they can take fish from fishing lines up to 1000 m depth^42^, biologging data suggest that they spend most of their time at <20 m depth^41^. Further, the protracted and intense pack hunting effort required for killer whales to subdue cetaceans^21^, and their restricted ~10 min dive duration^41^ strongly suggest that they can only hunt mammals at or near the sea-surface. We therefore propose that deep waters are a refuge where beaked whales are safe from killer whale attacks, and we predict that groups of beaked whales will coordinate their sound production and movements so as to minimise acoustic and visual detection when abandoning the deep refuge to return to the surface. We used two sources of data to test this notion: pairs of whales in the same social group were tagged simultaneously in three instances (two Cuvier’s pairs and one pair of Blainville’s) giving a complete quantification of their spatial relationship and coordination. Tagged whales were adults or subadults of both sexes swimming in larger social groups and we assume that their behaviour is a random sample of the behaviour of other group members. These data were supplemented with an analysis of movement patterns and inferred group vocal behaviour for a larger set of whales tagged individually, as well as an extensive dataset of sightings of both species.

**Figure 1:**
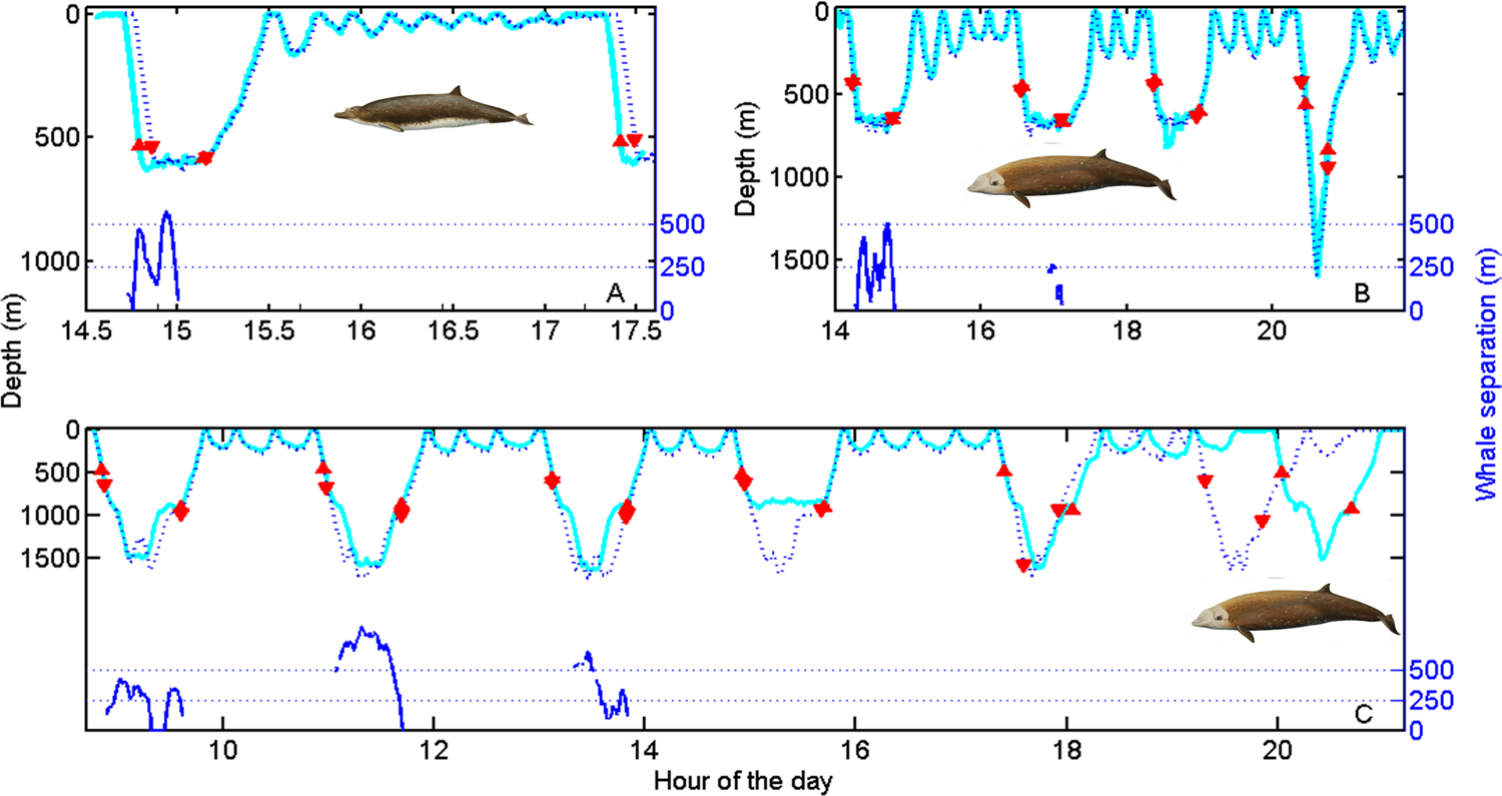
Dive profiles of three pairs of beaked whales tagged simultaneously in the same social group: (**A**) Blainville’s, Canary Islands; (**B**) Cuvier’s, Ligurian Sea and (**C**) Cuvier’s, Azores. For each pair, dive profiles are represented by cyan solid and blue dashed lines. Up and down red triangles mark when the first and second whale of the pair starts and finishes vocalising. The remarkable synchronization of dive and vocal activity of the whales while in a group (the Azorean group were observed to split at about 19:00), results in the whales being silent, and therefore largely undetectable by predators that rely on passive acoustics, some 80% of the time. Blue lines at the base of some dives indicate whale separation where this could be calculated from the travel time of the clicks emitted by one whale to the tag carried by its companion. Whales separate horizontally, and in one case vertically, by several hundreds of metres at the base of dives indicating individual foraging despite tight alignment of dive duration, ascent rate and vocal interval. Whale drawings by Brett Jarrett.

Using the data from paired tags, we analysed coordination in deep dives while the whale pairs remained in the same group. For each whale pair we identified the deep dives (i.e., >500 m) performed by the first tagged whale. For each such dive we then found the deep dive with closest start time performed by the second whale. This resulted in 10 deep-dive pairs for analysis (Fig. 1) with each deep dive of both whales associated with just one dive-pair. For these pairs of dives, the dive overlap, i.e., the proportion of the longest dive in each pair during which the other whale is also diving, averages 99% (SD 0.3%), while the overlap in the vocal phase, i.e., the part of the dive in which regular echolocation sounds are made, is 98% (SD 4%) (Table 1). One of the tagged pairs was in a group of Cuvier’s in the Azores which was observed to split after 9 hours, separating the tagged whales. The dive cycle recorded after this whale pair split had a dive overlap of only 29% while vocal overlap disappeared completely (Fig. 1). Table 1 reports the results of dive coordination for all whale pairs. For the Azores whale pair, the results are reported separately for the time before and after the whale pair split because from this point on the two whales were not in the same social group.

**Table 1:** Diving and vocal coordination of three pairs of whales tagged in the same social group for overlapping time periods.

Whale pair	# dive pairs	Max. depth (m)	Depth diff. (m)	Dur. (min)	Dur. diff. (min)	Total time overlap (%)	Vocal time overlap (%)
Blainville’s Canary Islands ♂♀	Deep n = 1	639	16	46	2	99	98
	Shallow n = 6	56 (37–108)	8 (2–13)	11 (9–14)	0.7 (0.5–1.2)	93 (91–96)	—
Cuvier’s Liguria Ind-Ind	Deep n = 4	954 (724–1600)	49 (6–135)	55 (48–64)	0.2 (0.2)	100 (99–100)	95 (90–100)
	Shallow n = 11	173 (114–275)	21 (6–42)	17 (15–20)	0.3 (0.1–1)	98 (94–100)	—
Cuvier’s Azores (pre-group split) Ind-Ind	Deep n = 5	1544 (1296–1669)	287 (89–769)	61 (60–62)	0.3 (0.1–0.6)	100 (100–100)	100 (100–100)
	Shallow n = 12	166 (142–189)	39 (22–61)	19 (16–22)	0.6 (0.2–0.8)	97 (95–98)	—
Cuvier’s Azores (post-group split) Ind-Ind	Deep n = 1	1574	134	61	0.1	29	0
	Shallow n = 3	270 (149–382)	65 (50–82)	19 (6–25)	7 (2–16)	42 (0–65)	—

For each pair of whales we located the deep dives performed by one whale and identified the deep dive of whale two with the closest start time. This resulted in 10 dive pairs including all deep dives of both whales. For these dives we quantified the time overlap and the vocal phase overlap (i.e., the minimum percent of time that one whale is diving/vocal when the other whale also is). Time overlap is computed separately for deep dives where whales forage using echolocation, and for silent shallow dives. Results are expressed as mean (range). Max depth: maximum depth of the paired dives; Depth diff: difference in maximum depth of the paired dives. Dur: dive duration. ♂ Male; ♀ Female; Ind: indeterminate sex.

To test if the tight synchronization observed in whales swimming in social groups could simply be a consequence of the highly stereotyped dive cycles of these species, we analysed the overlap between the real dive profile of one whale of each pair and a simulated profile obtained from the other whale by permuting its dive cycles (i.e., each deep dive plus the following series of shallow dives before the next deep dive). This analysis excluded the Blainville’s pair that only completed one dive cycle. All permutations of the two Cuvier’s whale pairs had lower dive and vocal overlap than the actual profiles, with permuted averages of 64% and 54% overlap, respectively. Close dive and vocal timing in whale pairs is therefore the result of active coordination among group members. This interpretation is supported by the immediate loss of coordination when the Azores whale pair split. It is also corroborated with a larger dataset by examining the vocal overlap of group members audible in tag sound recordings from 12 Blainville’s (46 deep dives) tagged separately in the Canary Islands (Table 2). The low ambient noise in this field-site means that presence/absence of echolocation clicks of group members can be reliably inferred. On average, group members began and ended clicking in deep dives within 1.8 (SD 1.5) and 0.9 (SD 1.0) min, respectively, of the tagged whales, giving an average vocal overlap of 99% of the mean 26 min long vocal phase of these dives. Thus, groups of beaked whales closely coordinate their deep dives resulting in almost complete overlap of the approximately five hours per day in which they produce sound to forage. This high vocal coordination means that groups of beaked whales are available for passive acoustic detection by eavesdropping killer whales less than 25% of the time, practically independent of group size.

**Table 2:** Difference in the timing of start and end of clicking (SOC and EOC, respectively) between individually tagged Blainville’s beaked whales and any untagged whale within acoustic range of the tags.

Whale	# vocal dives analysed	Duration vocal phase	Time-diff SOC	Time-diff EOC
Md03_284a	6	26.23 (4.9)	2.31 (1.21)	0.75 (1.34)
Md03_298a	2	24.79 (3.07)	0.05 (0.06)	0.35 (0.14)
Md04_287a	4	27.51 (4.22)	0.65 (0.8)	0.23 (0.21)
Md05_277a	3	25.38 (3.24)	2.03 (0.31)	1.06 (0.59)
Md05_285a	4	25.11 (2.18)	2.5 (1.63)	0.99 (1.17)
Md08_136a	2	24.32 (3.33)	0.73 (0.32)	0.39 (0.16)
Md08_137a	8	27.95 (5.87)	5.9 (4.74)	1.42 (0.78)
Md08_142a	1 of 2	20.42	1.82	0.26
Md08_148a	1 of 2	27.18	1.53	4
Md08_289a	7	26.18 (9.11)	1.82 (1.33)	0.73 (0.49)
Md10_146a	1	21.85	1.48	0.81
Md10_163a	7	20.5 (4.67)	0.75 (0.79)	0.2 (0.18)
mean (range)	n = 46	25.2 (20.4-30.5)	1.8 (0.05-2.56)	0.9 (0.22-4)

Results are given in minutes and expressed as the mean (SD) for each tag deployment. The name of the tag deployment is formed by the two last digits of the year, the Julian day of the deployment and a letter indicating the consecutive tag order of the day. All vocal dives were analysed except for the two indicated in which clicks from other animals could not be assessed due to elevated background noise (primarily flow noise on tags located posteriorly on the whale), or in which EOC could not be assessed because the tag released before the end of the dive.

A consequence of the close diving coordination is that beaked whales must limit their foraging durations to match those of other group members with potentially lower diving capacity, thereby reducing individual foraging efficiency. Beaked whales live in fission-fusion societies and form groups of individuals of different age groups and sizes which nonetheless coordinate their diving and surfacing times. Even young beaked whales are observed to dive along with adults: in 18 years of field observations in the field-site of El Hierro, Canary Islands, comprising some three thousand sightings of Blainville’s and Cuvier’s groups, young have consistently been observed to dive and re-surface in close coordination with adults. This is in contrast with other deep diving species that leave young at the surface under alloparental care of group members^43,44^ or whose calves perform shorter shallower dives, or both^45^.

The impact of coordinated diving on foraging efficiency might be less severe if beaked whales hunted as a pack, e.g., actively aggregating patches of deep prey. To test this possibility, we examined the separation distance during foraging dives between the three pairs of whales tagged in the same group, using the acoustic travel time of clicks between each pair to precisely track the animals. Whales were as close as 11 m (mean 154 m, SD 15 m, range 11–305) when they began echolocating at a mean depth of 450 m. They then separated by a mean of 287 m (SD 57 m, range 11–468 m) while hunting but closed in at the end of the vocal phase to a mean distance of 127 m (SD 15 m, range 28–297) (Fig. 1). These results are consistent with the diving behaviour of beaked whale groups inferred from acoustic tracking with hydrophone arrays^46^. Beaked whales therefore appear to separate to forage individually within dives but are constrained by the need to approach group members before they ascend to the surface in silence. Thus, beaked whale groups are in effect joined by an acoustic leash during deep dives limiting the total foraging footprint of the group to the distance over which group members can maintain acoustic contact and reunite during a carefully timed ascent. This coordination may benefit beaked whales if they monitor acoustically successful group members to guide prey search, but coordinating may also have foraging disadvantages. Beaked whales attempt to hunt some 20–30 prey per dive^2–5,23^. This means that a group of e.g. five whales diving in synchronicity need to find some 100-150 prey in 20-30 min of echolocation within an area defined by the detection distance of their clicks.

The consistently high diving and vocal coordination demonstrated by both tagged whale pairs and individually tagged whales within groups, covering two species and different geographical areas, strongly suggest that collective behaviour is critical for social beaked whale groups: although the obligate deep vocal foraging intervals put beaked whales at risk of detection and stalking by killer whales performing passive acoustic tracking, beaked whales are safe to vocalize while in their deep refuge and their collective diving behaviour frees them from the need to vocalize during ascents to re-join non-diving members at the surface. This is in contrast to pilot whales or sperm whales that often vocalize during ascents to mediate group reunion acoustically^10–13^. That beaked whales of different genera (Mesoplodon, Ziphius) show the same coordinated behaviour suggests that the coordination of diving and vocal activity in social groups may have evolved millions of years ago or has had sufficient evolutionary value as to drive convergence towards a strikingly similar strategy.

### Silent ascent swimming

Although tight vocal overlap reduces the acoustic detectability of beaked whales, they nonetheless face the risk that eavesdropping predators are waiting for them when they return to the surface. Compared to terrestrial mammals that must choose between refuges and foraging, beaked whales live in a through-the-looking glass world: they are safe while making sound to forage at depths beyond the reach of killer whales but are at maximum risk when they come to the surface to breathe. Unlike most prey species^47^, for beaked whales foraging is not risky but breathing is.

The low-angled powered ascents that are a distinctive feature of the foraging dives of Cuvier’s and Blainville’s^2^ have been proposed to serve in managing decompression sickness^6^ but the wide variability in overall ascent vertical speed from dive-to-dive (0.3-1.1 m/s Md, 0.4-0.9 m/s Zc^48^) is difficult to reconcile with a physiological need for a particular decompression rate. However, low pitch angle ascent swimming confers the direct advantage that beaked whales can cover a substantial horizontal distance during their silent ascents, potentially moving them away from waiting predators. We therefore hypothesise that beaked whales will move horizontally during ascents in such a way as to make it difficult for shallow diving killer whales tracking echolocation clicks produced at depth by beaked whales to predict where will they surface. Such a strategy is only possible for beaked whales because they do not need to re-join non-diving members at the surface. For other deep diving species such as pilot whales and sperm whales, non-diving group members, including calves left alone or under alloparental care at or near the surface^43–45^, form a surface anchor to which diving animals must return. In contrast, the collective behaviour of beaked whales frees them to choose their surfacing location.

**Figure 2:**
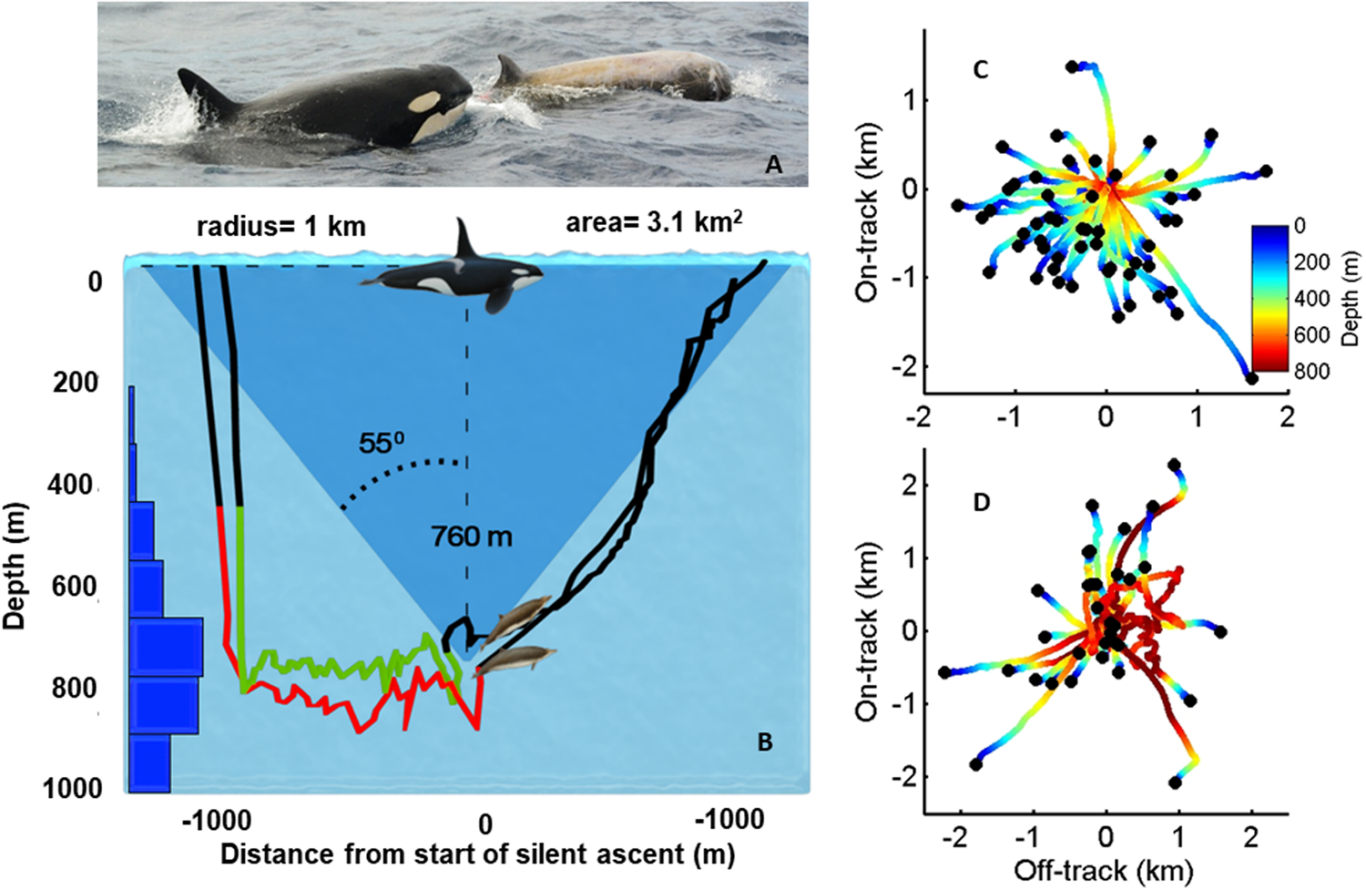
(**A**) Killer whales hunting a Cuvier’s beaked whale (photo by Machi Yoshida, Naturaliste Charters Australia). (**B**) Foraging dive tracks of two Blainville’s beaked whales tagged in the same group showing their activity synchronization. Coloured segments indicate hunting by echolocation whereas black segments indicate silent travel. Blue bars on the left show the depth distribution of all clicks from 14 tagged Blainville’s beaked whales, confirming that they are silent at depths shallower than 200 m where killer whales restrict most of their dives. Cuvier’s and Blainville’s beaked whales begin a silent ascent at a mean of 760 m depth and ascend with a shallow angle (mean 35° from the horizontal)^2,48^ in an unpredictable but coordinated direction. Dead-reckoned tracks show that ascending whales cover on average of 1 km horizontal distance from where they stopped clicking until they reach the surface, as represented schematically by the blue cone in panel B. (**C,D**) Horizontal dead-reckoned travel paths (coloured lines) of ascending Blainville’s and Cuvier’s beaked whales, respectively, with respect to their swimming direction before silencing. Travel in the same direction as the animal was moving prior to silencing is represented by the positive on-track axis in these plots while movements orthogonal to this are represented by the off-track axis. Surfacing positions (black dots) that are further from the centre of the plot are less predictable for an eavesdropping predator at the surface.

To test the predictability of beaked whale travel during ascents we estimated dead-reckoned tracks^40^, constructed from the pitch, roll, heading and depth data recorded by the DTAG, for 64 and 37 silent ascents of 14 Blainville’s and 10 Cuvier’s, respectively. These tracks were plotted with respect to the mean heading during the last five minutes of vocal activity before initiating the silent ascent. The resulting tracks show that beaked whales frequently adopt headings that translate them away from the surfacing position that would be predicted by an eavesdropping predator (Fig. 2C,D). On average, whales covered one kilometre horizontal distance from the point where they stopped clicking until they reached the surface (SD 430 and 710 m for Blainville’s and Cuvier’s respectively). This behaviour creates a large circular locus of potential surfacing positions that must be searched by killer whales and which they must search visually rather than using echolocation to avoid alerting their prey^24^.

The average 1 km horizontal distance covered by beaked whales in silence during the ascent renders a surfacing uncertainty circle with an area of 3.1 km^2^ (Fig. 2B) which waiting killer whales must search within the 2.5 min that Cuvier’s and Blainville’s spend on average at the surface after a deep vocal dive. Assuming a swimming speed of 2 m/s for killer whales^25^ and a maximum visual detection range in most oceans of some 50 m underwater^49^, an individual killer whale can cover visually only some 1.2% of the potential surfacing area of beaked whales during a surfacing. Encounter probability increases with killer whale group size: groups of up to 12 whales have been observed attacking beaked whales^21^ (Fig. 2a) which could still only cover some 15% of the potential surfacing area of beaked whales with a perfectly coordinated search pattern. Thus, even if killer whales detect beaked whale echolocation clicks acoustically, the unpredictable low-angle silent ascents decrease predator encounter rate by one to two orders of magnitude compared to the vertical ascents made by other deep diving whales^8,9^. But for this strategy to work, and even just to maintain group cohesion, beaked whale group members must ascend with similar speed and direction without additional vocal cues. This adds to the critical importance of tight coordination at the end of the vocal foraging interval.

In the intervals between deep foraging dives, beaked whales maintain almost complete silence and so likely need occasional visual contact with other members of their social group to maintain cohesion. Between deep dives these whales typically perform a sequence of shallow non-foraging dives^2,14,50^, which can nonetheless reach 400 m depth and 25 min duration, and in which animals can move hundreds of metres horizontally^2^. We predicted that group cohesion should be evident as strong synchrony in the dive profiles of group members in the extended intervals between deep dives. To test this, we used again a permutation method on paired dive profiles. Shallow silent dives performed by the three whale pairs overlapped in duration by an average of 97% (SD 2.4, n = 29 paired dives). In contrast, the overlap of 3000 simulated shallow dive profiles (1000 per whale pair) constructed using dives randomly selected from the same whale pair, averaged just 30% (SD 4). This is consistent with field observations of Blainville’s and Cuvier’s (El Hierro), and from the tagged Cuvier pair in the Azores (Fig. 1), where beaked whale groups tend to maintain close temporal and spatial cohesion in surfacing and diving, while coordination is lost when groups split.

### The costs of hiding

The collective vocal and diving behaviour of beaked whales greatly reduces both the time intervals over which groups can be detected by acoustic predators, and the post-detection interception risk, in effect enabling beaked whales to hide from eavesdropping predators. Although there may be additional benefits of close vocal and movement coordination, e.g., in sharing foraging information via mutual acoustic monitoring, as has been observed in echolocating bats^51^, this synchronization comes at a significant cost. The long silent ascents reduce the time available for foraging by some 35% as compared to vertical ascents, the common strategy of other deep-diving cetaceans^8,9^, for the same dive duration. Moreover, the closely synchronized diving behaviour must accommodate group members across a range of diving capacities, further constraining the foraging time of larger individuals. This perhaps explains the unusually large size of newborn beaked whales (approx. 50% length of the mother)^52^ in comparison to other toothed whales which are born at about one third of the adult size. A large birth length, and therefore weight, likely confers an advanced start for the ontogenetic development of diving capabilities and favours juveniles rapidly attaining the diving performance needed to dive with adults. Such large birth size may also explain why female beaked whales are similar in size or larger than males^52^ despite inter-male fights that would be expected to drive sexual dimorphism towards larger males. Similar body size may have the further benefit of harmonising diving capacity among group members reducing the cost of accommodating diverse diving endurance within a group.

### Conclusions and conservation implications

The unique diving and vocal behaviour of beaked whales could only evolve if the severe costs it imposes are outweighed by survival benefits. While the natural social and diving behaviour of beaked whales may be influenced by a whole suite of physiological, life history and ecological factors, we show here that the features that make beaked whale diving and vocal behaviour distinctive compared to other toothed whales confer major quantifiable advantages in abating predation risk from killer whales and even from visual predators at the surface such as sharks. These results provide the first quantitative support for previous hypotheses that the behaviour of beaked whales is influenced by predation risk^2,4,14^. Thus, while sperm whales and pilot whales, aided by either size or numbers, can choose to stay and fight off killer whale attacks^28,29,53^, beaked whales with little social defence have adopted the strategy of hiding. Unlike many terrestrial prey species navigating landscapes of fear^54^ for which risk assessment is modulated temporally by perception of the state of predators and indirect predation cues^15,16^, beaked whales have little opportunity to assess risk, as mammal eating killer whales tend to hunt silently^24^ and can only be seen at short range underwater^49^. As a consequence, for beaked whales, tonal sounds above ambient noise that might signal killer whale presence or other threats could well provoke an anti-predator response^32–35^. The beaked whale strategy of hiding is borne out in their responses to sonar and killer whale playbacks: silencing and avoidance^32–35^. Evolution in a soundscape of fear therefore offers a mechanistic explanation for why beaked whales respond so strongly to playbacks of sonar and killer whale sounds at barely audible levels. Akin to ungulate escape responses from pursuing predators that can lead to death by physiological stress^55^, naval sonar that inadvertently signals a strong risk-factor, such as the sounds of apex predators, may push beaked whales beyond their physiological limits and in some cases lead to sonar induced mortalities. As such, a successful predator abatement strategy shaped by natural selection has become maladaptive in the face of novel human activities. Given the vast zones over which mid-frequency navy sonars are audible and so may impact the behaviour of beaked whales^32–35^, large-scale spatial avoidance of beaked whale habitats when mid-frequency sonar is used should provide the most effective mitigation measure for these cryptic species^56^.

## Methods

### Data collection

Beaked whales were studied using suction-cup attached DTAGs^40^ containing depth and orientation sensors (3-axis accelerometers and magnetometers) sampled at 50 or 200 Hz and two hydrophones sampled with 16-bit resolution at 96, 192, or 240 kHz. Blainville’s beaked whales (n = 14), were tagged off El Hierro (Canary Islands, Spain^3,4^); Cuvier’s beaked whales were tagged in the Gulf of Genoa (Ligurian Sea, Italy^2^), n = 10, and off Terceira (Azores, Portugal,), n = 2. Whales were approached slowly from a small boat and the tag was deployed on the back of the whales with the aid of a handheld pole. Tags remained attached for up to 20 hours and were located for recovery using VHF tracking after their release from the whales.

### Analysis of tag data

Tag data were analysed in Matlab (www.mathworks.com). Depth and movement data were calibrated with standard procedures^40^. Sound recordings were examined as in previous analysis of beaked whale DTAG data^2–4,23^ with custom tools from the DTAG toolbox (www.soundtags.org) to identify vocalizations of the whales. Vocalizations comprised echolocation clicks and buzzes^23^, as well as rasps and more-rarely whistles with an apparent communication function^4^. Echolocation clicks were located individually with the aid of a supervised click detector^23^.

Cuvier’s and Blainville’s beaked whales perform deep and long foraging dives (deeper than 500 m) interspersed with series of shallow dives defined as dives between 20 and 500 m depth^2^. Surfacing intervals separating consecutive dives (i.e. deeper than 20 m) lasted on average 2.5 min (SD 0.6) and 2.6 min (SD 1.3) for Cuvier’s and Blainville’s beaked whales, respectively (mean of the median duration of the surface intervals performed by each whale, grouped by species).

When echolocating in deep foraging dives, beaked whales produce 2-3 clicks per second with occasional buzzes and short pauses. The vocal phase was defined as the interval in which this regular clicking and buzzing takes place in a deep dive^2,23^.

### Coordination of diving and vocal behaviour

Diving behaviour was analysed as in previous analysis of beaked whale DTAG data^2–5^. Groups of beaked whales were defined as clusters of whales observed together at the surface. No inferences were made about short or long-term group stability. Whales in these clusters were most often observed to surface together for the duration of the visual follow, albeit groups can also split. On three occasions (one per field site) we succeeded in tagging two whales in the same social group. Tag deployments on the two members of these whale-pairs overlapped in time during 3, 9 and 12 hours; the 6 whales forming these whale-pairs performed in total 22 deep and 64 shallow dives.

The *separation distance* between whales in each tagged whale pair was estimated during the vocal phases. This was achieved by measuring the time delay between the emission of a click by one tagged whale and the reception of the same click on the tag carried by the other whale in the pair. Comparison of time delays for clicks produced by each of the two whales allowed for estimation of the clock offset between the two tags. Clock offset was subtracted from the measured time delays to give the acoustic time of flight which was then converted to distance by multiplying by the path-integrated sound speed. Depth profiles of sound speed for each location were used together with the known depths of each animal to derive the path-integrated sound speed for each click. Sound speed profiles were gathered from CTD (RBR Ltd. and Sea-bird Scientific Inc.) deployments performed in El Hierro and the Ligurian Sea at the time of tagging, and from the AZODC database for Azores (http://oceano.horta.uac.pt/azodc/oceatlas.php) in a relatively close area and season of the year with respect to the tagging event.

### Ascents from deep dives

Blainville’s and Cuvier’s beaked whales only forage during the deeper part of deep dives. In these dives they end echolocation at a mean depth of 738 m (Blainville’s) and 856 m (Cuvier’s). Descents in deep dives are performed with a pitch angle close to 90° and a vertical speed of 1.5 m/s (Blainville’s) and 1.6 m/s (Cuvier’s). However, both species ascend with a low pitch angle (approx. 35°) and a vertical rate of about 0.7 m/s (0.7 ± 0.1 Blainville’s and 0.8 ± 0.15 Cuvier’s)^48^. This means that whales take on average 9 min (Blainville’s) and 11.5 min (Cuvier’s) longer in ascending than if they were to ascend with the same speed and vertical posture that they use in descents. Given the average vocal phase duration of 26 min (Blainville’s) and 33 min (Cuvier’s)^2^, the extended duration of the ascent represents about 35% of the duration of the foraging time for both species.

### Surfacing uncertainty area due to slow ascents

To assess the predictability of horizontal movements during ascents, dead-reckoned tracks^40^ were computed for the ascent (i.e., from the end of vocal activity until the whale reached the surface) of each deep dive of tagged whales for which the orientation of the tag on the animal could be estimated reliably, resulting in 52 and 33 ascent tracks from 14 Blainville’s and 10 Cuvier’s. As the DTAG lacks a speed sensor, the average orientation-corrected depth rate (OCDR)^8^ over each ascent was used as a speed proxy in dead-reckoning. The OCDR is sensitive to noise at low pitch angles and so speed values were omitted from the average when the absolute pitch angle was less than 20 degrees. The ascents tracks were rotated by the negative of the average heading during the final five min of vocal activity in each dive so that an ascent track that continued in the same direction as the whale was moving while vocalizing would have a heading of 0°. We considered that horizontal movements taking the ascending whale away from the point of last vocalization would be increasingly unpredictable for an eavesdropping predator at the surface. The average surfacing location predicted from the dead-reckoned tracks was horizontally offset by 999 m (S.D. 434 m) and 1019 m (S.D. 709 m) from the last vocal position in Blainville’s and Cuvier’s, respectively (Fig. 2C,D), with the tracks largely moving away from the direction of travel that the animals held in the last 5 min of vocal activity. This horizontal movement of a mean of 1 km in any direction gives a potential surfacing circle with area 3.1 km^2^ (Fig. 2B) that would need to be searched visually by a predator to locate beaked whales that had been tracked acoustically while diving.

### Ethics and permissions

All experiments were performed in accordance with relevant guidelines and regulations for studying wild animals. Data were gathered with ethics authorization of Woods Hole Oceanographic Institution Animal Care and use Committee, the University of La Laguna Animal Use Ethics Committees and the KMR Institutional Animal Care and Use Committee. Research permits were granted by the US NMFS permits 981-1578-02 and 981-1707-00 (for data gathered at Italy) and the Governments of Spain and the Canary Islands, and by the Secretaria Regional do Mar, Ciência e Technologia, Direção Regional dos Assuntos do Mar (permit number 10/2015/DRA, for Azores).

## References

[1] Fedak MA, Thompson D (1993). Behavioural and physiological options in diving seals. Symposium Zoological Society of London.

[2] Tyack PL, Johnson M, Aguilar de Soto N, Sturlese A, Madsen PT (2006). Extreme diving of beaked whales. J. Exp. Biol..

[3] Arranz P (2011). Following a foraging fish-finder: diel habitat use of Blainville’s beaked whales revealed by echolocation. PLoS One.

[4] Aguilar de Soto N (2012). No shallow talk: Cryptic strategy in the vocal communication of Blainville’s beaked whales. Mar. Mammal Sci..

[5] Madsen PT, Aguilar de Soto N, Tyack P, Johnson M (2014). Beaked whales. Curr. Biol..

[6] Hooker SK (2012). Deadly diving? Physiological and behavioural management of decompression stress in diving mammals. Proc. R. Soc. B Biol. Sci..

[7] Velten BP, Dillaman RM, Kinsey ST, McLellan WA, Pabst DA (2013). Novel locomotor muscle design in extreme deep-diving whales. J. Exp. Biol.

[8] Miller PJO, Johnson MP, Tyack PL, Terray EA (2004). Swimming gaits, passive drag and buoyancy of diving sperm whales Physeter macrocephalus. J. Exp. Biol..

[9] Aguilar Soto N (2008). Cheetahs of the deep sea: deep foraging sprints in short-finned pilot whales off Tenerife (Canary Islands). J. Anim. Ecol..

[10] Jensen F, Marrero J, Johnson M, Aguilar de Soto N, Madsen PT (2011). Calling under pressure: Short-finned pilot whales make social calls during deep foraging dives. Proc. R. Soc. B..

[11] Marrero J, Jensen F, Rojano L, Aguilar de Soto N (2017). Different modes of acoustic communication in deep diving short‐finned pilot whales (Globicephala macrorhynchus). Mar. Mam. Sci..

[12] Visser F (2017). Vocal foragers and silent crowds: context-dependent vocal variation in Northeast Atlantic long-finned pilot whales. Behavioral Ecology and Sociobiology.

[13] Oliveira C (2016). Sperm whale codas may encode individuality as well as clan identity. J. Acoust. Soc. Am..

[14] Baird RW, Webster DL, Schorr GS, McSweeney DJ, Barlow J (2008). Diel variation in beaked whale diving behavior. Mar. Mamm. Sci..

[15] Zanette L, White A, Allen M, Clinchy M (2011). Perceived predation risk reduces the number of offspring songbirds produce per year. Science..

[16] Creel S, Christianson D (2008). Relationships between direct predation and risk effects. Trends Ecol. Evol..

[17] Ford J, Reeves R (2008). Fight or flight: antipredator strategies of baleen whales. Mammal Rev..

[18] McSweeney DJ, Baird RW, Mahaffy SD (2007). Site fidelity, associations and movements of Cuvier’s (Ziphius cavirostris) and Blainville’s (Mesoplodon densirostris) beaked whales off the island of Hawai’i. Marine Mammal Science.

[19] Cañadas, A. pers.comm. Observations of striped dolphins harassing a Cuvier’s beaked whale in the Mediterranean.

[20] Jefferson TA, Stacey PJ, Baird RW (1991). A review of killer whale interactions with other marine mammals: predation to co-existence. Mammal Rev..

[21] Wellard R (2016). Killer whale (Orcinus orca) predation on beaked whales (Mesoplodon spp.) in the Bremer Sub-Basin, Western Australia. PLoS One.

[22] Gualtieri D, Pitman RL (2019). Killer Whale (Orcinus orca) Predation on a Gervais’ Beaked Whale (Mesoplodon europaeus) in the Eastern Atlantic Ocean. Aquat. Mamm..

[23] Johnson M, Madsen PT, Zimmer WMX, Soto NAde, Tyack PL (2004). Beaked whales echolocate on prey. Proc. R. Soc. London. Ser. B Biol. Sci..

[24] Barrett-Lennard LG, Ford JKB, Heise KA (1996). The mixed blessing of echolocation: differences in sonar use by fish-eating and mammal-eating killer whales. Anim. Behav..

[25] Williams R, Noren DP (2009). Swimming speed, respiration rate, and estimated cost of transport in adult killer whales. Mar. Mammal Sci..

[26] Andersen, S. & Amundin, M. Possible predator-related adaption of sound production and hearing in the harbour porpoise (Phocoena phocoena). Aquat. Mamm (1976).

[27] Morisaka T, Connor RC (2007). Predation by killer whales (Orcinus orca) and the evolution of whistle loss and narrow-band high frequency clicks in odontocetes. J. Evol. Biol..

[28] De Stephanis R., Giménez J., Esteban R., Gauffier P., García-Tiscar S., Sinding M-H. S., Verborgh P. (2014). Mobbing-like behavior by pilot whales towards killer whales: a response to resource competition or perceived predation risk?. acta ethologica.

[29] Curé C (2013). Responses of male sperm whales (Physeter macrocephalus) to killer whale sounds: implications for anti-predator strategies. Sci. Rep..

[30] Marques TA, Thomas L, Ward J, DiMarzio N, Tyack PL (2009). Estimating cetacean population density using fixed passive acoustic sensors: An example with Blainville’s beaked whales. J. Acoust. Soc. Am..

[31] Baird, R. W. Behavior and Ecology of Not-So-Social Odontocetes: Cuvier’s and Blainville’s Beaked Whales. In: Würsig (ed.), *Ethology and Behavioral Ecology of Marine Mammals*. Springer. ISSN: 2523–7500 (2019).

[32] Tyack PL (2011). Beaked whales respond to simulated and actual navy sonar. PLoS One.

[33] Miller PJO (2015). First indications that northern bottlenose whales are sensitive to behavioural disturbance from anthropogenic noise. R. Soc. Open Sci..

[34] Allen AN, Schanze JJ, Solow AR, Tyack PL (2014). Analysis of a Blainville’s beaked whale’s movement response to playback of killer whale vocalizations. Mar. Mammal Sci..

[35] DeRuiter SL (2013). First direct measurements of behavioural responses by Cuvier’s beaked whales to mid-frequency active sonar. Biol. Lett..

[36] Frantzis A (1998). Does acoustic testing strand whales?. Nature.

[37] Jepson PD (2003). Gas-bubble lesions in stranded cetaceans. Nature.

[38] Bernaldo de Quirós Y (2019). Advances in research on the impacts of anti-submarine sonar on beaked whales. Proc. R. Soc. B.

[39] D’Amico A (2009). Beaked whale strandings and naval exercises. Aquat. Mamm..

[40] Johnson MP, Tyack PL (2003). A digital acoustic recording tag for measuring the response of wild marine mammals to sound. IEEE J. Ocean. Eng..

[41] Miller PJO, Shapiro AD, Deecke VB (2010). The diving behaviour of mammal-eating killer whales (Orcinus orca): variations with ecological not physiological factors. Can. J. Zool..

[42] Towers J (2019). Movements and dive behaviour of a toothfish-depredating killer and sperm whale. ICES J. Mar. Sci..

[43] Whitehead H (1996). Babysitting, dive synchrony, and indications of alloparental care in sperm whales. Behav Ecol Sociobiol.

[44] Augusto, J. F., Frasier, T. R. & Whitehead, H. Characterizing alloparental care in the pilot whale (Globicephala melas) population that summers off Cape Breton, Nova Scotia, Canada. *Mar. Mammal Sci* (2016).

[45] Tønnesen, P., Gero, S., Ladegaard, M., Johnson, M. & Madsen, P. T. First-year sperm whale calves echolocate and perform long, deep dives. *Behav. Ecol. Soci*. **72**(10), 10.1007/s00265-018-2570-y (2018).

[46] Gassmann M, Wiggins S, Hildebrand J (2015). Three-dimensional tracking of Cuvier’s beaked whales’ echolocation sounds using nested hydrophone arrays. JASA.

[47] Clinchy M, Sheriff M, Zanette L (2013). The ecology of stress. Predator-induced stress and the ecology of fear. Func. Ecol..

[48] Martin Lopez LM, Miller PJO, Aguilar de Soto N, Johnson M (2015). Gait switches in deep-diving beaked whales: biomechanical strategies for long-duration dives. J. Exp. Biol..

[49] Berman T, Walline P, Schneller A, Rothenberg J, Townsend D (1985). Secchi disk depth record: a claim for the Eastern Mediterranean. Limn. Oceano..

[50] Baird RW (2006). Diving behaviour of Cuvier’s (Ziphius cavirostris) and Blainville’s (Mesoplodon densirostris) beaked whales in Hawai’i. Can. J. Zool..

[51] Barclay RMR (1982). Interindividual use of echolocation calls: Eavesdropping by bats. Behav. Ecol. Sociobiol..

[52] Mead, J. G. Beaked whales, overview. In: Encyclopedia of Marine Mammals, 2nd edition. Perrin, W. F., Würsig, B. & Thewissen, J. G. M. (Eds.), Academic Press, San Diego, pp. 81–84 (2002).

[53] Pitman R, Balance L, Mesnick S, Chivers S (2001). Killer whale predation on sperm whales: observations and implications. Mar. Mam. Sci..

[54] Laundré J, Hernández L, Ripple W (2010). The landscape of fear: ecological implications of being afraid. Open Ecol. J..

[55] Blumstein D (2015). The evolution of capture myopathy in hooved mammals: a model for human stress cardiomyopathy?. Evolution, Medicine, and Public Health.

[56] Fernández, A., Sierra, E., Martin, V. M. M. & Sacchini, S. Last ‘atypical’beaked whales mass stranding in the Canary Islands (July, 2004). *Journal Marine Science Research and Develpment* **2** (2012).

